# Focus on neuroenhancement: a systematic review and its ethical implications

**DOI:** 10.1192/j.eurpsy.2022.913

**Published:** 2022-09-01

**Authors:** L. Massa, S. Palermo, T. Ivaldi, A. Della Vecchia, F. Mucci, D. Marazziti, L. Dell’Osso

**Affiliations:** 1 University of Pisa, Department Of Clinical And Experimental Medicine, Pisa, Italy; 2 University of Siena, Department Of Biotechnology, Chemistry And Pharmacy, Siena, Italy

**Keywords:** Ethics, neuroenhancement, emotional enhancement, psychopharmacology

## Abstract

**Introduction:**

Pharmacological and cognitive neuroenhancement refer to the non-medical use of prescription drugs, alcohol, illegal drugs, or the so-called soft enhancers, to enhance cognition, mood, work or school performance, or to promote pro-social behaviour. Literature on the topic is meagre, and available data only partially enlightens their use.

**Objectives:**

The aim of this paper is to review and comment on the available literature on pharmacological neuroenhancement and, secondary, on emotional enhancement.

**Methods:**

A systematic review was conducted according to the PRISMA guidelines. Pubmed, Scopus, Embase, PsychInfo and Google Scholar databases were accessed to select English language articles, published from 1980 to April 2020. 11746 papers were initially selected and 123 papers were finally included.

**Results:**

Available literature indicates a widespread and increasing use of different kinds of substances, drugs and food supplements mainly with neuroenhancing purposes, especially amongst specific populations of young healthy subjects. The evidence regarding their efficacy is controversial. Further, a limited or no awareness regarding the possible consequences of their abuse/misuse emerges amongst users.

**Conclusions:**

Despite the limited evidence that some substances may improve cognitive functions in healthy subjects and neglecting their detrimental side effects and potential risk of misuse, abuse and addiction, there is an increasing worldwide use of the so-called neuroenhancers, especially in some categories of individuals, such as university students. Further studies are needed to collect reliable data on the effects of neuroenhancers in healthy subjects. Neuroenhancement puts into question the concept of authenticity, so that the problem requires to be analyzed within a complex ethical conceptual frame.

**Disclosure:**

No significant relationships.

